# Caries Ossium Cranii ex Syphilitide

**Published:** 1813-03

**Authors:** A. B. Granville

**Affiliations:** South Audley-street, Grosvenor-square


### Medical and Physical Journal.

3 OF VOL. XXIX.] MARCH, 1813. [NO. 169.

*To the Editors of the Medical and Physical Journal.*

GENTLEMEN,

IN the course of my last services in the Mediterranean, as surgeon of His Majesty's ship Swiftsure, one of those cases which seldom occur to professional men attached to the navy, fell under my observation. It was a case of disease in some of the bones of the cranium—a disease which too often terminates unfavorably, but which, in this instance, took the most propitious turn.

The faculty have been compelled, at various and distinct periods of the history of medicine, to acknowledge, with much regret, the very limited acquaintance they had with the diseases of the head. Never was confession made with more justice. Affections of the head are so many and various, that, when they do not proceed from accident, and their origin cannot be traced from external violence, we are left to guess at their nature without a guide, or at the utmost with that only of a few well-authenticated precedents. Diseases affecting the bony structure of the head, fall, of course, under this same observation. When violently struck, contused, or divided, the morbid state of the head admits of close inspection; we undertake its treatment on sure grounds, and we are expert in it. But, when we discover it to be morbidly affected, without being able to trace or ascertain the cause, then we are obliged to pause. We consult our greatest authorities in vain: no assistance is to be derived from them—no friendly hint to be obtained—we are left to speculation; and surely, amidst the many unpleasant situations for a professional man, this dubious state of the mind is the most distressing.

James Courtenay, aged 33, a petty officer, formerly an active seaman, and of a disposition particularly sober, had been for a considerable time under treatment for certain wandering pains, chiefly affecting the long bones and the large joints, and of course supposed to be rheumatic. At one time, indeed, the surgeon whom I had succeeded had ventured to characterise them as venereal, and had begun a treatment accordingly; but, to the insinuation associated with this mode of viewing the disease, the patient resisted with some firmness—"He was a married man, and had not been affected by disease of any kind for the long period of twelve years."—In a mind naturally virtuous, even in the lower classes of society, any suspicion tending to affect their correctness of conduct, excites becoming indignation. Sailors are not aware that the effects of debauchery can be felt at a considerable distance of time; and, as the patient, in this instance, was conscious of not having wandered from domestic intercourse, he thought it due to his own happiness, and that of his partner, not to admit the idea to which the suggestions of his medical attendant seemed to give rise. The surgeon yielded to the remonstrance, and the disease was treated as a rheumatic affection.

At this period (23d March, 1812,) I joined the Swiftsure. The patient seemed then emaciated and weak; his countenance was pale and dejected; he complained of severe pains affecting various parts of his body, but chiefly the long bones; his pulse beat 60, and distant. I confess I made no particular inquiry at the moment, and ordered the continuation of those medicines which he had been in the habit of taking, with some trifling addition of others from the class of STIMULI. Several days passed without much or any alteration, when the obstinacy of his arthritic complaint roused my more particular notice. I examined him strictly, and, having gained sufficient reason to believe that he was laboring under the impression of venereal virus, I treated him accordingly.

After the elapse of a few days, the patient, for the first time, mentioned "having a swelling on his head." On inspection, I found a considerable elevation of the scalp, occasioned by the collection of some substance, which felt grumous at the touch, yielding and elastic, without however any sensible fluctuation. The lower circle of the swelling seemed to be formed as if the subjacent bones had been perforated and their edges raised. At one time I even felt, or fancied I could feel, a degree of pulsation in the tumor. I inquired of my patient whether he had ever received any injury in the head at that or at any other time; but to this he steadily answered in the negative. "He experienced some shooting pains across the forehead; was extremely light in the head; but the *swelling gave him no uneasiness.* He had only been sensible of it the preceding night." I felt perplexed. Was this an abscess? but, if so, how formed? We had no evident cause to ascribe it to, nor did the contents appear to be the produce of, suppuration. Could it be a tumor, properly called, connected or not with the internal internal parts of the head? A fungus,* for example, an osteosarcosis, or a steatoma? † I had seen an instance of cephalic tumor while attending clinical lectures, and a celebrated surgeon had treated it with pressure. Other instances are recorded by Le Dran, and pressure is also recommended. I determined to act on *safe* principles, and applied a thick piece of sheet-lead, in the form of an inverted cup, which I fixed and pressed gently down by a double-headed roller. At the end of three days I perceived my deception; I therefore passed a large seton-needle into the most prominent part of the swelling; no pus, however, followed; there came away a few drops of blood, and the swelling remained unaltered. I then passed my directory through the opening, and made an incision of two inches transversely over the tumor, by which I separated the integuments sufficiently to discover that the tendinous expansion of the occipito-frontalis formed the *capsule* of the abscess. A large lancet was struck deep into this, when about four ounces of pus came away, together with some parts of the diseased pericranium and occipito-frontal aponeurosis. The wound was cleaned, and the extent of the disease ascertained. The two parietal bones, where they are joined by the sagittal suture, just on the "vertex capitis," were carious to the extent of more than one inch each side; and, on lengthening the incision, further portions of the same bones appeared denuded of their *pericranium*. Dossils of lint, impregnated with a strong solution of sulphate of copper, were used to

---

* Richerand, in his Surgical Nosography, speaking of the diseases by him called vegetations of the dura mater, observes, "that there are some fungous tumors, which, arising from the external surface of the superior meninge, elevate themselves through the bones of the cranium, after having previously destroyed them by their action." After remarking that these are generally the effects of violent strokes on the head, severe falls, &c. he proceeds: "Even the venereal virus, ‡ which any where else produces periostosis, can in this case give origin to tumors of this description."

† Monteggia, in a philosophical work on surgery, asserts having seen several cases of osteosarcosis, which he describes as a soft tumor of the bones, sometimes affecting those of the head, and always produced by the total want of the phosphate of lime. It has a sensible pulsation, which he calls "pulsazione communicata," (when the osteosarcosis occupies the bones of the head,) being a pulsation communicated to the tumor from distant arteries, in contradistinction to "pulsazione organica," which names he gives to the pulsation occasioned by arteries passing through the affected part.

‡ La Lue e lo scorbuto sono le principali cause di questa malattia.—*Conradi Anatomica Patologica*, 1806.

 dress dress the cavity left by the abscess during two consecutive days, when, the patient appearing to lose strength rapidly, his pulse beating low and small, his appetite gone, and the wound assuming no better aspect, I resolved to remove the whole of the diseased part of the bones by operation. This was performed on the 12th of April. An incision was made to part from the former one, which on this occasion was likewise lengthened, so as to give to the whole the figure of a T. The bones having been denuded as far as necessary, I penetrated with the largest trephine (which encompassed the greatest portion of the diseased parts) into and beyond the meditullium, when, with the assistance of the *bone-nippers*, I removed the whole of the carious portion of the bones, leaving the internal table, which appeared to be healthy, intact. There were some other *straggling* impressions of *caries* at some distance from the principal spot, and these I destroyed by means of a sharp-cutting instrument not unlike a *graver*. The wound was dressed with oleaginous substances, its edges were brought together, and kept in that state by crucial straps of adhesive plaister, over which a compress was applied, with decoction of bark. A night-cap, fixed by a handkerchief, formed the bandage. The patient, who had borne the tedious operation with uncommon fortitude and patience, and who seemed much exhausted, was remanded to bed, and had an opiate draught given him. In the evening 12 ℥ of blood were taken from the arm, and at bed-time a course of mercurial treatment was begun, so calculated as to excite pthyalism quickly.

From this period (12th April) to the 18th, the disease alternately promised well and threatened further mischief. Salivation was soon obtained and kept up; the external wound assumed the most healthy aspect, but the bones still appeared affected. The caries baffled all my attention; for, no sooner had I destroyed it in one spot, when it made its appearance at another, penetrating and perforating, at last, even the internal table, which had been left sound after the operation. Pulsation now became visible through the corroded bone, the pain in the forehead increased to a violent degree, the pulse was scarcely perceptible. There was extreme defection of strength, vomiting, fœtid diarrhœa, stupor, and coma. The scene was about to be closed, I ordered a draught of equal parts of Sp. Lav. comp. and water, with xx drops of Tinct. Opii, to be given every hour during the night, in hopes merely of keeping life in until the succeeding morning, when, if the strength of the patient would allow it, I intended repeating the operation of trepanning, as the last effort to save him. On the 21st, a small head-saw was used on on that spot of the internal table, within the first trepanned circle, which the caries had now eroded; a considerable portion of it was removed, the meninge discovered, and thus a free exit given to the pus collected on its surface. The more distant portions of diseased bone were also removed with the same instrument mentioned in the first instance of the operation; and, to change the morbid action that might still continue to affect the bony structure of the head, nitrate of silver was applied to that part of it immediately discovered by, and connected with, the disease.* The opening was afterwards defended with lint dipped in oil of olives, and the whole of the wound had likewise soft dressings applied to it. *Curatio altera ut antea.*

The change brought on by this last effort to save our patient, was as prompt as it was propitious. It almost exceeded my best expectation. The pulse rose to strength immediately after the operation; the coma and stupor were relieved by the removal of the pus from off the superior meninge; and hope once more cheered the resigned sufferer.

From this instant his convalescence may be dated. The pain in the forehead, after being much relieved by the application of blisters to the temples, disappeared at last. Sleep, which had been purchased by large doses of opium, now occurred natural, regular, and easy. Appetite returned. Strength, keeping pace with health, diffused itself throughout the system, and he finally quitted his bed.

During few subsequent weeks, I had the gratification of witnessing the progress of renovated ossification. Granulations shooted forth in every direction from the innermost

---

* I am sorry to find a man of so well-earned celebrity as Richerand revive, and with the warmest recommendation support, the abolished practice of using actual cauterium to destroy the caries of spongious bones. He says, "The most energetic caustic can be used according to the profundity of the caries; when the sanies is abundant, the actual cautery is even indicated; the pus that issues from the carious bones diluting the caustic applications, soon extinguishes their power. It is thus that the red-hot iron offers the advantage of destroying the part at the same moment of its application; of turning the *caries* into *necrosis*, and of arresting most efficaciously the progress of the disease."—"I caustici più energici potranno essere usati secondo la profondità della carie; quando la sanie abbonda è indicato perfino il cauterio attuale; il pus che sgorga dall osso cariato rilavando i caustici extingue ben presto il loro potere. Il ferro rovente offre allora il vantaggio di bruciare nell' istante medesimo dell' applicazione, di ridurre sollecitamente la carie in necrosi ed arrestarne efficacemente i progressi."—*Richerand, Nos. Chir. Ediz. Ital.*

circle of the orifice in the bones, and proceeded upwards, luxuriant and rapid. Exfoliation, at the same time, took place in those few spots where the nitrate of silver had been applied.

The wound finally cicatrised in June, about 70 days from the first moment of the disease being ascertained. It is to the deficiency of part of the scalp caused by the disease, that the tedious length of time elapsed before the ultimate healing of the integuments is to be ascribed.

I must not forget to mention that, during the most unfavorable period of the disease, a tumor appeared amongst the fibres of some of the cervical muscles of the right side, no doubt from sympathetic absorption. It felt as the original swelling had on the head. I disregarded it, and it disappeared gradually from the first moment of the original disease improving.

I forbear from all comments on the above case; and I conclude with simply remarking, that from it I have learned never to abandon to nature a local affection, however unfavorable, whilst a moment's reflection can suggest *any*, even the least promising, expedient for the final cure of it.

I have the honor to be, &c. &c.

*South Audley-street,* A. B. GRANVILLE, M.D. &c.

*Grosvenor-square.*

